# Highly oxidized albumin is cleared by liver sinusoidal endothelial cells via the receptors stabilin-1 and -2

**DOI:** 10.1038/s41598-023-46462-9

**Published:** 2023-11-05

**Authors:** Christopher Holte, Karolina Szafranska, Larissa Kruse, Jaione Simon-Santamaria, Ruomei Li, Dmitri Svistounov, Peter McCourt

**Affiliations:** 1 Vascular Biology Research Group, Department of Medical Biology, UiT The Arctic University of Norway, Tromsø, Norway; 2 Metabolic and Renal Research Group, Department of Clinical Medicine, UiT The Arctic University of Norway, Tromsø, Norway

**Keywords:** Biochemistry, Cell biology, Molecular biology, Physiology, Medical research, Pathogenesis, Risk factors

## Abstract

Oxidized albumin (oxHSA) is elevated in several pathological conditions, such as decompensated cirrhosis, acute on chronic liver failure and liver mediated renal failure. Patient derived oxidized albumin was previously shown to be an inflammatory mediator, and in normal serum levels of oxHSA are low. The removal from circulation of oxidized albumins is therefore likely required for maintenance of homeostasis. Liver sinusoidal endothelial cells (LSEC) are prominent scavenger cells specialized in removal of macromolecular waste. Given that oxidized albumin is mainly cleared by the liver, we hypothesized the LSEC are the site of uptake in the liver. In vivo oxHSA was cleared rapidly by the liver and distributed to mainly the LSEC. In in vitro studies LSEC endocytosed oxHSA much more than other cell populations isolated from the liver. Furthermore, it was shown that the uptake was mediated by the stabilins, by affinity chromatography-mass spectrometry, inhibiting uptake in LSEC with other stabilin ligands and showing uptake in HEK cells overexpressing stabilin-1 or -2. oxHSA also inhibited the uptake of other stabilin ligands, and a 2-h challenge with 100 µg/mL oxHSA reduced LSEC endocytosis by 60% up to 12 h after. Thus the LSEC and their stabilins mediate clearance of highly oxidized albumin, and oxidized albumin can downregulate their endocytic capacity in turn.

## Introduction

Albumin is the most abundant protein in blood (40 g/L of plasma is made up of albumin^1^, and it has a correspondingly large number of functions, including binding and transporting a host of ligands including but not limited to free fatty acids, drugs (including: warfarin, salicylic acid, propofol, lidocaine) and metabolites^2^. In the blood stream albumin serves as the main antioxidant due to its readily reacting cysteine-34 residue and metal ion binding properties, and is also found extensively in the extravascular extracellular space^2,3^. Albumin is therefore a vital component in mitigation of oxidative stress throughout the body.

Oxidative stress is implicated in the pathophysiology of several diseases, such as atherosclerosis; where oxidation products are linked with plaque formation^4^; nephrotic damage in leukocyte-dependent glomerulonephritis^5^; and the development and progression of neurodegenerative diseases^6^. Ischemia-modified albumin, thought to be formed by reaction with reactive oxygen species and or hydroxyl radicals^7^, therefore a form of oxidized albumin, is a marker of poor prognosis in patients reporting chest pain, and is determined clinically by assaying the cobalt binding ability of patient sera^8^. The neutrophil myeloperoxidase is one endogenous system capable of producing extremely potent oxidants such as hypochlorite, thio- and hypothiocyanite^9^, thus serving as a link between inflammation and oxidative stress.

Oxidative stress and the presence of oxidized albumin is also a component of the pathogenesis of acute on chronic liver failure^10,11^, a syndrome that develops from decompensated cirrhosis^12^. Elevated advanced oxidation protein products (AOPP) and modified albumins were also found in plasma samples from idiosyncratic drug-induced liver injury^13^. Oxidation of serum albumin in patients with cirrhosis and bacterial peritonitis causes decreased binding properties of albumin, predicting impaired transport function^14^. In vitro oxidized albumin has been shown to have altered affinities, both increased and decreased, to various drugs and metabolites^15,16^.

Oxidised albumin is associated with a number of other pathologies. There is a correlation between the fraction of oxidized albumin and atherosclerosis development^17^. In nephrotic patients oxidized and advanced glycation end-product (AGE) albumin was found, and a reduction in oxidized albumin considered a beneficial marker after hemodialysis^18^. The oxidation products themselves have been suggested to be uremic toxins playing an active role in the development of chronic renal failure^19^. An increased fraction of oxidized relative vs. non-oxidized albumin is also characteristic of Diabetes Mellitus patients^20^. Oxidized albumin from hypoalbuminemic hemodialysis patient samples were shown to cause elevated expression of inflammatory cytokines in HUVECs^21^ and primary peripheral blood leukocytes^22^. The proinflammatory effect was shown to be oxidation dependent and reversible upon chemical reduction of the albumin^21^. Oxidative modifications of HSA have been shown to induce clearance from circulation^23^, showing a potential link between hypoalbuminemia and oxidative stress often observed in cirrhosis. Iwao^24^ found chemically oxidized HSA (oxHSA), produced using the hypochlorite analogue chloramine-T, to be similar to oxidized albumin found in uremic patients. This oxHSA was found to be rapidly cleared from circulation in mice, primarily by the liver (51%) and spleen (23%), which are two of the major scavenging organs in the body.

Liver sinusoidal endothelial cells (LSEC) are known to take up a host of macromolecular waste from the bloodstream^25^ whereas Kupffer cells (KC), the liver resident macrophages, remove larger (> 200 nm) complexes from the circulation^26^. Modified albumins such as Advanced Glycation End-products-BSA (AGE-BSA) and formaldehyde modified BSA (FSA) are taken up by the liver sinusoidal endothelium^27–29^, the scavenging endothelium of the liver sinusoids. AGEs^30,31^ FSA^27,32,33^ were shown to be primarily endocytosed via the scavenger receptor class H^34^ (SR-H), also known as stabilin-1 and -2. Oxidized low density lipoproteins oxLDL^35^ and acetylated LDL^36^ were also shown to be taken up by the liver sinusoidal endothelial cells via stabilin-1 and -2. Stabilin-1/2 double knockout (KO) mice exhibit glomerular fibrosis, with significant reduction to the animal lifespan, indicating that a reduction in clearance via the stabilins in the liver had downstream effects on the kidneys^37^.

The stabilins are further implicated in the development of several pathologies, either caused by deficiency/insufficiency or for atherosclerosis where they seem to contribute to plaque formation. KO models of stabilin-1 or -2 showed decreased atherosclerotic plaque formation under Western diet conditions or ApoE KO^38,39^—the effect was replicated using monoclonal antibodies and suggested as a therapy against atherosclerosis in prone individuals, as antibodies would likely not greatly interfere with liver endothelial scavenging^39^. Stabilin double KOs exhibit transforming growth factor beta induced protein (TGFBI) and Periostin (POSTN) deposition in liver and in glomeruli with age^40^. Even single KO models were found to have increased inappropriate deposition of connective tissue components, and showed more severe steatosis and fibrosis in induced models^41^. Stabilin-1 was shown in a mouse model to be protective against viral myocarditis, with stabilin-1 KOs showing worse inflammation in the heart^42^. Gene correlation analysis and mouse model studies showed that stabilin-2 deficiency was associated with a prothrombotic phenotype^43^. Stabilin-2 is found to be highly expressed in cells surrounding atherosclerotic lesions in a mouse Ldlr KO model, and was suggested as a way to target these^44^. Stabilin double KO mice exhibited significant placental abnormalities, and produced few viable offspring, this was likely due to the reduced clearance of apoptotic cells during placental remodeling^45^. The liver and spleen are the main sites of stabilin 1 and 2 expression^46,47^.

The ability to induce oxidative stress-like damage of oxidation protein products combined with the rapid uptake of oxHSA by the liver and the propensity for LSEC to clear modified proteins, make these cells a potential site of clearance of oxidized albumin and of injury during sustained oxidative stress.

If oxHSA binds to a scavenger receptor, as its rapid clearance suggests, this may have implications for the clearance of other waste molecules by the same. We therefore sought to determine which cell type and receptor take up oxHSA in the liver and describe the effects of oxHSA upon these cells.

## Results

### In vivo biodistribution

**Figure 1:**
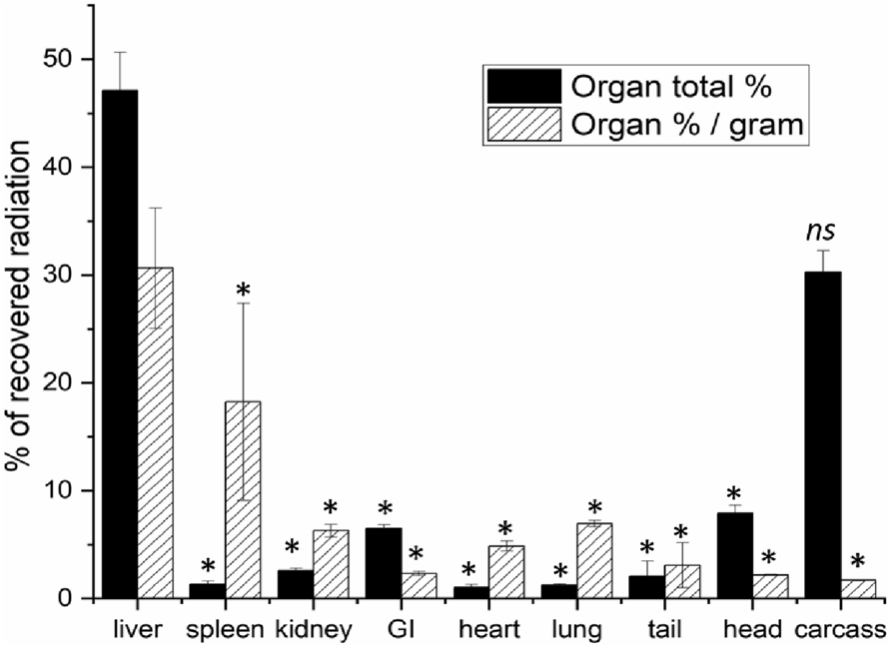
Biodistribution of oxHSA. 1–5 µg ^125^I radio-labelled oxHSA was injected intravenously, and animals were sacrificed 30 min post-injection. Uptake is given as % of total recovered radioactivity (black bars) or as % of total recovered radioactivity per gram of organ (white/shaded bars). Results are given as averages ± standard deviation over bio replicates, n = 3 animals, ns = not significant, * = p < 0.05 compared with liver (Independent Samples Median Test).

The liver took up the majority of injected oxHSA of all the organs with on average 47% of total radiation, 6.5% GI-tract, 8% head, 2.6% kidneys, 2% tail, 1.3% spleen, 1.2% lungs, 1% heart and 30% remained in the carcass. The liver and spleen took up the most radioactivity per mass, 30% and 18% per gram respectively (Fig. 1).

The majority of the injected (75%) radiation (as estimated from injected dose or total radiation in organs + carcass) was cleared before the first blood sample was collected 0:55–1:40 min post injection (Figure S2). Therefore, the t_1/2_ is even lower (< 90 s).

### Hepatocellular distribution

**Figure 2:**
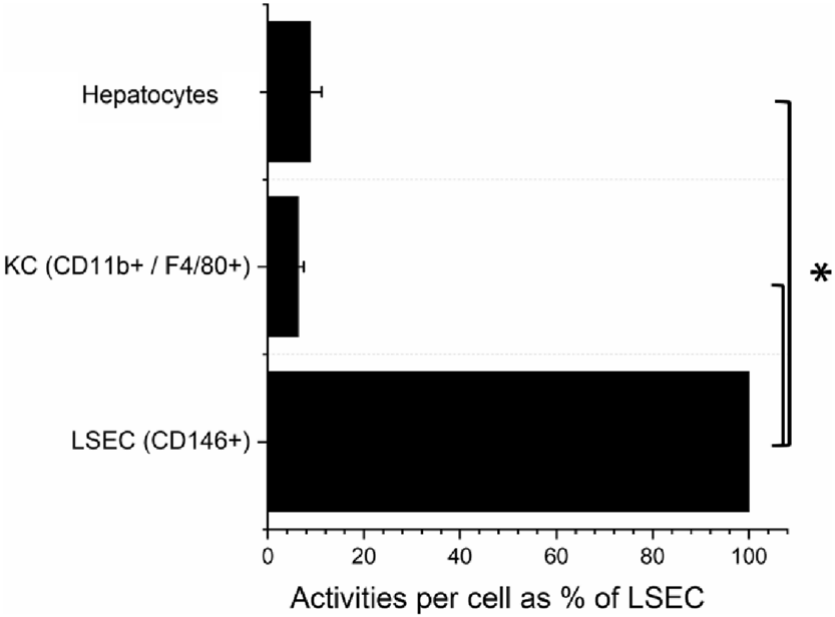
Hepatocellular distribution of oxHSA. Animals were injected with 1–5 µg ^125^I radio-labelled oxHSA, sacrificed 5 min post-injection and LSEC, Kupffer cells and hepatocyte fractions were isolated. Graphs show radioactivity per cell normalized to LSEC, in isolated fractions of liver cells (selected by; CD146:LSEC, CD11b & F4/80:Kupffer cells, Percoll 45%:hepatocytes). Results are given as averages ± standard deviation over bio replicates, n = 3 animals, * = p < 0.05 (Independent Samples Median Test).

To determine the relative contribution of liver cells to oxHSA hepatocellular distribution was performed. Out of the cells of the liver the LSEC had the highest activities, compared to Kupffer cells or hepatocytes. LSEC contained activites (normalized to cell number) 15 and 11-fold higher than KC or hepatocytes respectively (Fig. 2).

### In vitro uptake in isolated liver cell populations

**Figure 3:**
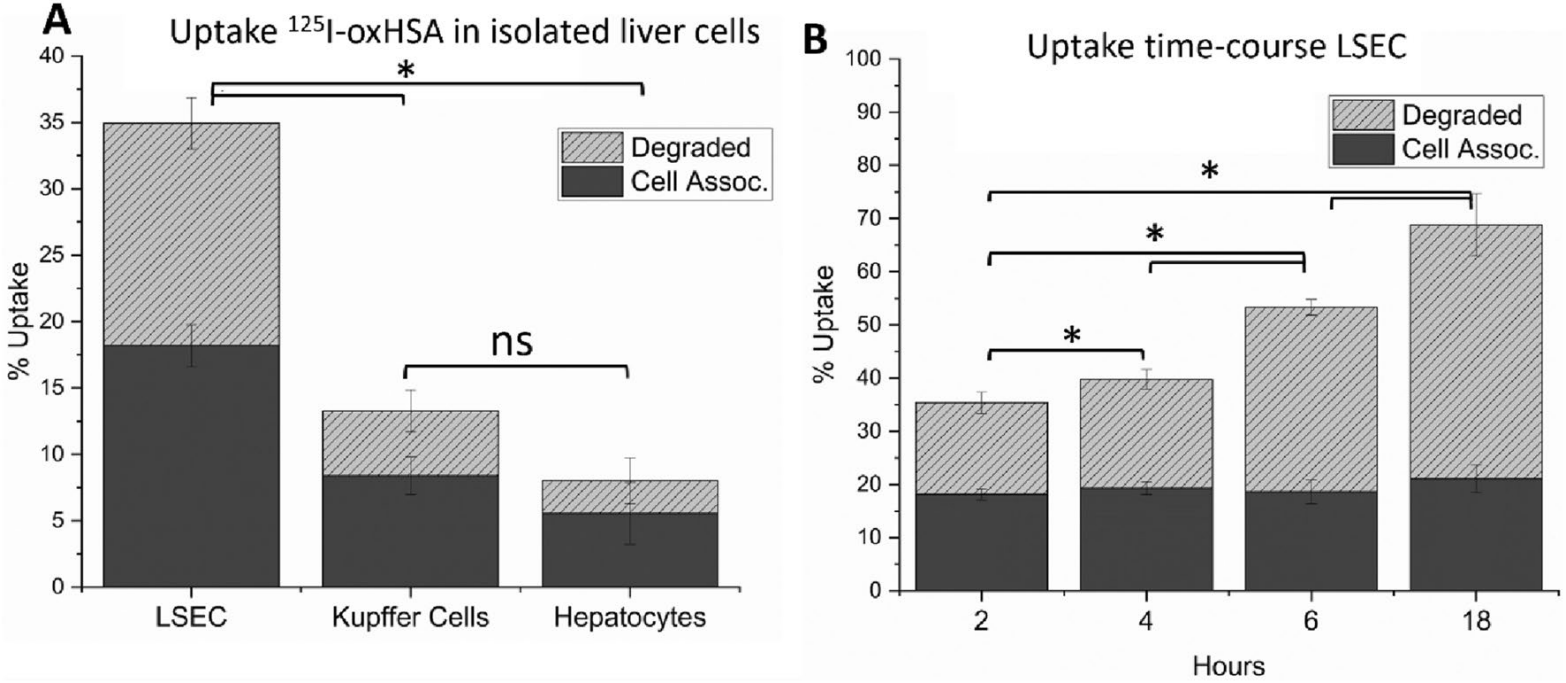
In vitro uptake of oxHSA by isolated liver cells. (**A**) Uptake of ^125^I-oxHSA per 300 K cells in LSEC, Kupffer cells and hepatocytes. LSEC and hepatocytes were seeded 300K/well, Kupffer cells were counted and uptake calculated per 300K cells. (**B**) Time-course of ^125^I-oxHSA uptake in LSEC. Uptake is given as % of added (approx. 5-15ng/well). Solid bars indicate cell associated radioactivity, shaded bars indicate acid soluble radioactivity (= degraded ligand). Results are given as averages ± standard deviation over bio replicates, n = 3 animals, ns = not significant, * = p < 0.05, (Independent Samples Median Test (**A**), Independent Samples Jonckheere-Terpstra Test (**B**)).

Isolated murine LSEC showed the highest in vitro uptake with 35% uptake and degradation of added ^125^I-oxHSA over 2 h of incubation increasing to 70% after 18 h, per 300 K cells (Fig. 3A, B). Kupffer cells (resident macrophages) took up ≈ 13% of added ^125^I-oxHSA per 300 K cells over 2 h (Fig. 3A). Hepatocytes took up ≈10% of added ^125^I-oxHSA per 300 K cells over 2 h, but this likely due to contamination by NPCs (Fig. 3A).

### In vitro identification of the oxHSA endocytosis receptor

LSEC detergent lysates were subjected to affinity chromatography on oxHSA coupled to Sepharose. A number of proteins were eluted from this column, including stabilins-1 and -2. Stabilins-1 and -2 were not eluted from control columns; i.e. Sepharose without protein, or Sepharose coupled with native HSA. No other scavenger class receptors were eluted from the column. Importantly, the cell lysates contained all cellular proteins, and not only cell-surface proteins (Supplementary Table 1, Supplemental-MS).

**Figure 4:**
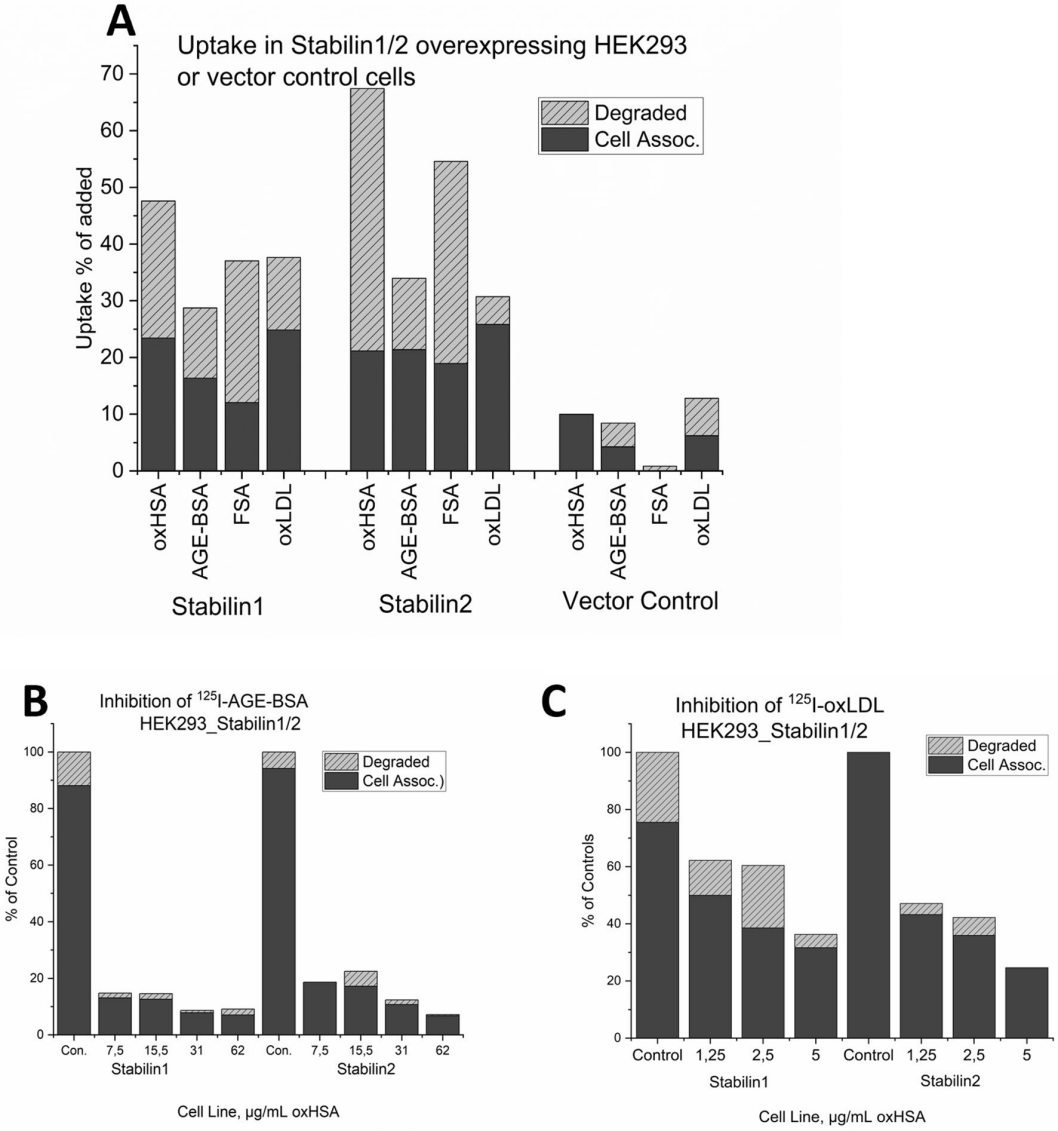
Uptake & competitive inhibition in HEK cells expressing stabilin-1 or -2. (**A**) Uptake of ^125^I-labelled oxHSA compared with uptake of other ligands for stabilin-1 and -2 (AGE-BSA, FSA, oxLDL) in HEK293 cells expressing stabilin-1, -2, or transfected with the empty vector. (**B**) Inhibition of ^125^I-AGE-BSA uptake in stabilin-1 and -2 expressing HEK cells by oxHSA. (**C**) Inhibition of ^125^I-oxLDL uptake in stabilin-1 and -2 expressing HEK cells by oxHSA. Uptake (in **A**) is given as % of radioactivity added per well, for inhibition graphs (**B-C**) uptake is given as % of (untreated) controls.

To determine the potential role of the SR-H scavenger receptors stabilin-1 and stabilin-2, HEK293 cells stably over expressing mouse stabilin-1 and stabilin-2 were challenged with ^125^I-oxHSA. Both stabilin-1 and stabilin-2 HEK293 cells (but not the empty vector control) avidly endocytosed 48% and 67% of trace amounts of ^125^I-oxHSA, respectively, within 4 h (Fig. 4A). Figure 4A shows the % endocytosis of added ^125^I-oxHSA to the abovementioned HEK293 cells, as well as other known SR-H ligands: FSA; AGE-BSA and oxLDL. These other ligands were endocytosed at 29–32% and 32–55% by stabilin-1 and stabilin-2 HEK293 cells, respectively. The empty vector control cells endocytosed ≤ 12% of added ligand.

The specificity of SR-H mediated uptake of oxHSA was tested by using oxHSA to inhibit uptake of other SR-H ligands. Stabilin-1 and stabilin-2 HEK293 cells were incubated with ^125^I-AGE-BSA (Fig. 4B) or ^125^I-oxLDL (Fig. 4 C) and challenged with unlabelled oxHSA (0–62 μg/ml or 0–5 μg/ml, respectively). ^125^I-AGE-BSA uptake was markedly (80% reduced relative to controls) inhibited in both SR-H expressing HEK293 cells at 7.5 μg/ml oxHSA. ^125^I-oxLDL uptake in the same cells was somewhat (60–70% reduced relative to controls) inhibited with 5.0 μg/ml oxHSA.

**Figure 5:**
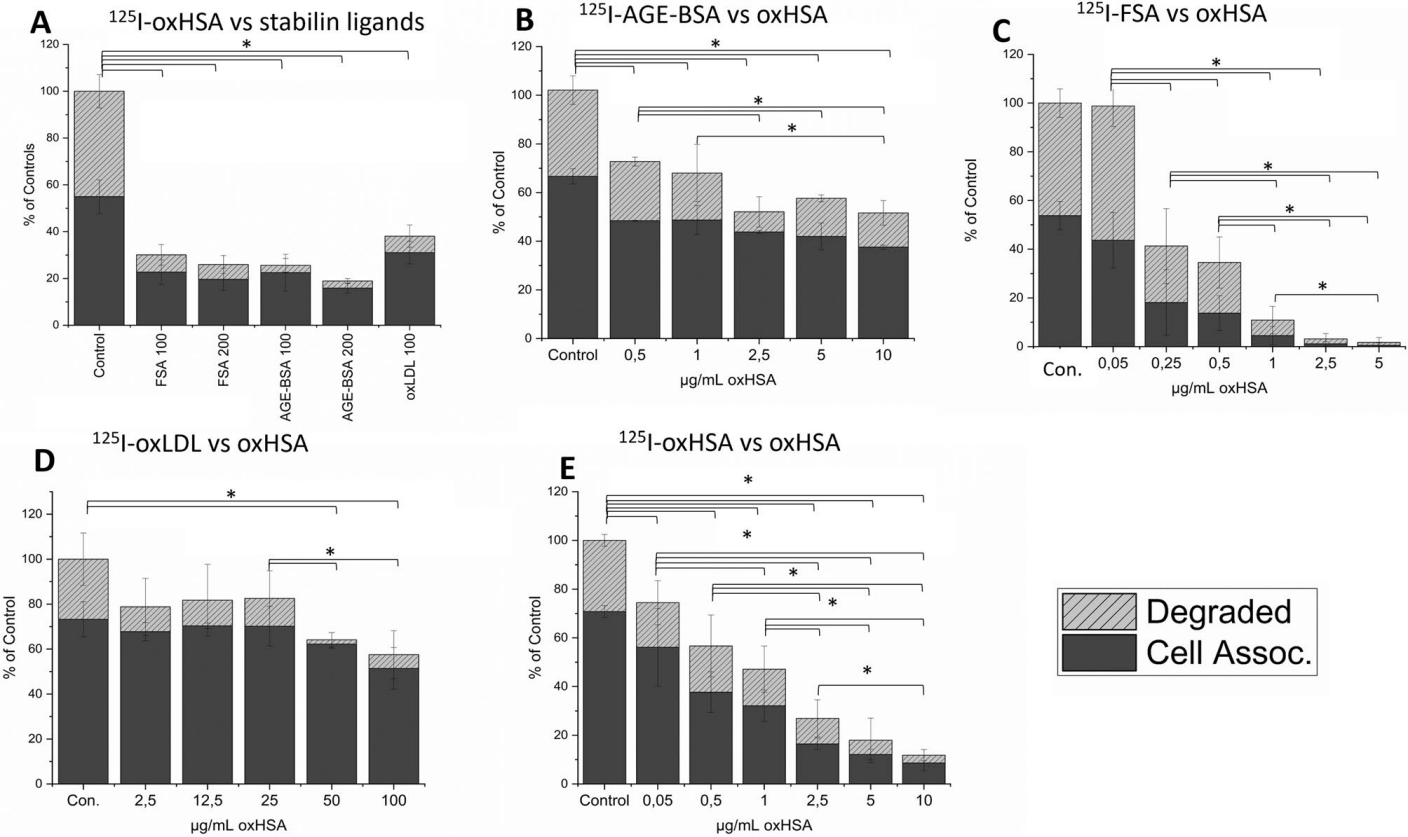
Competitive inhibition studies in LSEC. (**A**) Inhibition of ^125^I-oxHSA uptake in LSEC by other ligands of stabilin-1 and -2 (FSA, AGE-BSA, oxLDL). (**B**) Inhibition of ^125^I-FSA uptake in LSEC by oxHSA. (**C**) Inhibition of ^125^I-AGE-BSA uptake in LSEC by oxHSA. (**D**) Inhibition of ^125^I-oxLDL uptake in LSEC by oxHSA. (**E**) Inhibition of ^125^I-oxHSA uptake in LSEC by unlabelled oxHSA. Uptake is given as % of (untreated) controls. Solid bars indicate cell associated radioactivity, shaded bars indicate acid soluble radioactivity (= degraded ligand). Results for are given as averages ± standard deviation over bio replicates, n = 3 animals, *:p < 0.05, (Independent Samples Median Test (**A**), Independent Samples Jonckheere-Terpstra Test (**B-E**)).

Similar uptake and inhibition studies were performed on LSEC, which express both stabilin forms. LSEC challenged with 10 µg/mL Alexa488-oxHSA for 30 min showed marked uptake as determined by fluorescent microscopy (Figure S3). AGE-BSA, FSA and oxLDL inhibited the LSEC uptake of ^125^I-oxHSA by 60–80% relative to controls (Fig. 5A). Unlabeled oxHSA inhibited the LSEC uptake of ^125^I-FSA (50–90% reduced relative to controls), ^125^I-AGE-BSA (30–50% reduced relative to controls) and ^125^I-oxLDL (20–40% reduced relative to controls) (Fig. 5B–D). Unlabeled oxHSA markedly (25–90% reduced relative to controls) inhibited LSEC uptake of ^125^I-oxHSA (Fig. 5E), but not to the same degree as it did with FSA (Fig. 5C).

### Recovery of endocytosis

**Figure 6:**
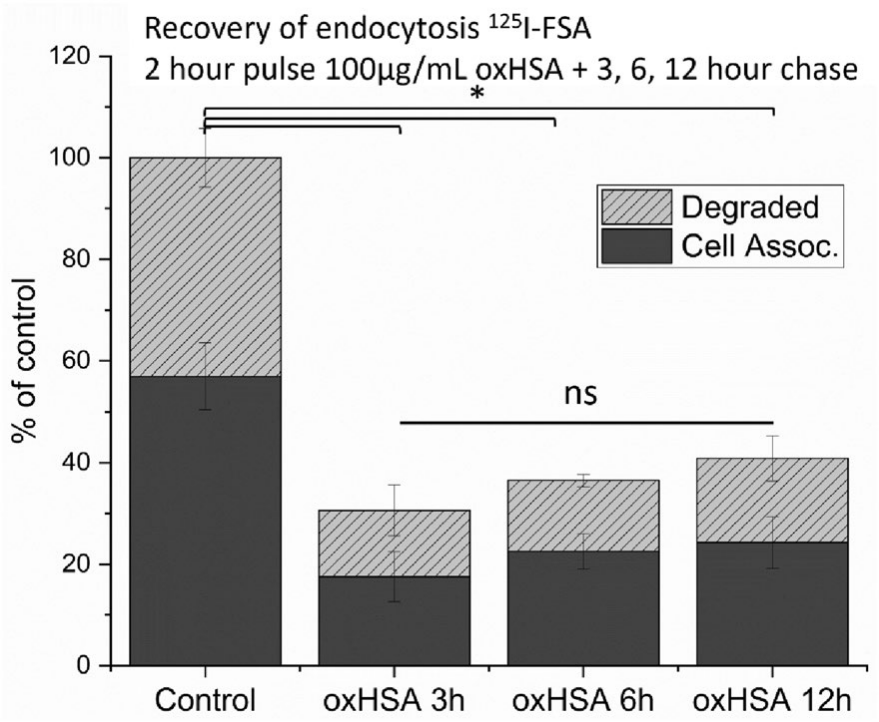
Pulse-chase/Recovery of endocytosis in LSEC. LSEC were treated with 100 µg/mL oxHSA × 2 h, and then the indicated number (3, 6, or 12) of hours chase in cell culture media, before endocytosis experiments with ^125^I-FSA. Uptakes in % of matched untreated controls. Solid bars indicate cell associated radioactivity, shaded bars indicate acid soluble radioactivity (= degraded ligand). Results given as averages ± standard deviation over bio replicates, n = 3 animals, * = p = 0.025 (Independent Samples Jonckheere-Terpstra Test).

To determine if the oxHSA-mediated inhibition of LSEC endocytosis was short or long term, we determined the level of FSA endocytosis after a 2-h pulse of oxHSA (100 μg/ml) followed by chases of 3, 6 and 12 h in RPMI media (Fig. 6). There was little to no recovery of LSEC FSA endocytosis to control levels even after a 12-h pulse of media (Fig. 6) where levels were at 40% of untreated levels.

### Morphology and viability of LSEC challenged with oxHSA

LSEC treated with 10–160 µg/mL oxHSA for 1 h showed no morphological alterations at EM level (Figure S4). Cells treated with 0–320 µg/mL for 3–6 h showed no changes to viability as measured by LDH or resazurin assays (Figure S5).

## Discussion

AOPP albumin, also known as oxHSA, is cleared from the circulation primarily by the liver and spleen^24^. We synthesized oxHSA to determine the site of its uptake in the liver and its effect on liver cells. oxHSA characterization by HPLC revealed increased size peaks relative to HSA’s peak (Figure S1), this is indicative of conformational rearrangement rather than added mass, as the electrophoretic motility under denaturing conditions (SDS) do not show such dramatic changes^24^. The HPLC profile of oxHSA is furthermore a very similar profile to model ligand FSA (data not shown). These conformational changes predispose albumin to scavenger receptor mediated clearance, judging by the examples of oxHSA and FSA. The oxHSA produced in this study was not toxic for LSEC as determined by LDH and resazurin assays, and the morphology of the cells was also seemingly unaffected by oxHSA as judged by SEM (Figure S4). We show that of all the liver cells, LSEC show the highest capacity for clearance of oxHSA (Figs. 2, 3). The most likely candidate receptors mediating this process are the SR-H scavenger receptors stabilin-1 and -2. This would be consistent with the observation that the highest stabilin expression levels are in the liver and spleen^46^.

We established that oxHSA is cleared by stabilins-1 and -2 by uptake and competitive inhibition studies in LSEC and HEK293 cells constitutively expressing stabilin-1 and -2. Ideally this would have been further validated by silencing stabilins in LSEC in vitro, however LSEC endocytic activity gradually decreases over time, with 60–70% reduction in uptake after 48 h and 80–85% reduction in uptake after 72 hours^48,49^. This prevents determination if reduced endocytosis after silencing would be caused directly by silencing or from a reduction in endocytic activity. For LSEC uptake of oxHSA was inhibited by FSA, AGE-BSA and oxLDL, and oxHSA in turn inhibited their uptake (Fig. 5). The uptake of FSA was completely inhibited in LSEC by oxHSA, indicating a very similar binding profile. oxHSA moderately inhibited AGE-BSA in LSEC (Fig. 5) but inhibited AGE-BSA uptake very strongly in stabilin expressing HEK293 cells (Fig. 4). oxLDL uptake/degradation was slightly inhibited by oxHSA in LSEC but very strongly in stabilin expressing HEK293 cells (Figs. 4, 5), suggesting these ligands (AGE-BSA, oxLDL) have additional receptors for endocytosis in LSEC.

We performed pulse chase experiments to determine if the effect of oxHSA on endocytosis was long lasting. Endocytosis was reduced to 40% of controls 12 h after challenge with 100 µg/mL for 2 h (Fig. 6), which is comparable to previously described effects of AGE-BSA on endocytosis mediated by stabilins-1 and -2^50^. This suggests that oxHSA depletes binding activity over a physiologically relevant timeframe. Thus, circulating oxHSA may impair the clearance of other stabilin ligands, which may be of concern during pathological states with high oxidative stress. For example it has been shown that stabilins in the liver are responsible for the elimination of LPS arriving from the gut, preventing systemic inflammation^51^. It has previously been shown that SR-H deficiency causes kidney fibrosis in a mouse model^37^. This was also suggested as a link between diabetic AGE formation and diabetic reno-pathy, which also sees heightened levels of oxidation protein products^35^. Partial hepatectomy often leads to kidney injuries^52^, where a reduction in clearance of scavenger receptor ligands may be a driver of these injuries. This fits with the presence of oxidized albumin in uremic patients^24^ as either a marker for reduced clearance or a uremic toxicant itself.

Additionally, it has been shown that the scavenger endothelium of the liver is the main site of clearance for pro-atherogenic molecules such as oxidized LDL, and AGEs^35,53^. An increased circulation time, or accumulation of these ligands is likely to cause atherosclerotic plaques and localized inflammation in the vasculature. Oxidative stress and detection of oxidation protein products has been linked with atherosclerosis previously^4^, with AOPP-Albumin been shown to cause atherosclerotic plaque formation in rabbits^54^.

Stabilins themselves have been implicated in the pathogenesis of atherosclerosis, with amelioration of atherosclerosis development in stabilin KO models^38,39^, this is indicative that binding by stabilins is part of the pathogenesis of the condition. oxHSA has a very high affinity for stabilins (greater than FSA), from this we can hypothesize that oxHSA and other stabilin ligands may, in non-scavenger sites, cause or exacerbate deleterious effects such as for example atherosclerotic lesions. Binding to stabilins may facilitate attachment by circulating immune cells and initiate inflammatory responses at the site^55^. Further stabilin-2 was found to also be a cell signalling receptor, activating the MAPK/ERK signalling pathway^56^. The implications of this signalling via stabilins especially under pro-atherogenic, or oxidative stress conditions is not well understood. Both stabilin-1 and -2 were found to bind E. coli and S. aureus in vitro^57^, thus they may be involved in both attachment of immune cells, and of bacteria sensing in other non-scavenger cell types, given their signalling capabilities^56^. This could conceivably be part of the mechanism of stabilin mediated atherogenesis, with immune cell recruitment and inflammation caused by ligand activation.

The stabilins are also the receptors for clearance of apoptotic cells/ cell corpses and aged red blood cells^58,59^, that circulating stabilin ligands such as oxHSA could interfere with this process therefore seems likely.

This suggests a common theme and possible feedback mechanism for these ligands, where an increase above a threshold will lead to a vicious cycle, where AOPP clearance is inhibited by their own prevalence, and their prevalence induces their own formation by an oxidative stress/ inflammation related mechanism at the sites of deposition. Thus, atherosclerosis and systemic inflammation is both driving and being driven by AOPP formation. This would all have implications for other organs, such as kidneys, as suggested by Schledzewski et al.^37^.

Similarly, the pathogenic progression of liver disease or injury, would lead to a reduction in clearance of the atherogenic oxidation protein products (oxHSA, oxLDL etc.) as was indeed found by Öettl in 2013^14^, which would increase their relative concentrations, circulation time, leading to deposition, plaque formation and inflammation. Plausible mechanisms driving this would be the impaired synthesis of new albumin by hepatocytes coupled with impaired clearance of modified albumins from circulation by LSECs.

In summary oxHSA is cleared in vivo by the LSEC, is a ligand for stabilins-1 and -2, and in vitro challenge of LSEC with oxHSA causes downregulation of SR-H mediated endocytosis. This has implications for the clearance of waste proteins, LPS and other ligands normally cleared by SR-H, since elevated levels of oxidized albumin are seen in diseases such as atherosclerosis, diabetes and acute and chronic liver failure.

If oxidized albumin interferes with SR-H mediated clearance, this may explain some of the downstream effects of pathological inflammation. Strategies inhibiting the formation of oxidation protein products during disease and inflammation may thus be warranted. Interventions such as those reviewed by Forman and Zhang 2021^60^ may be of use in such cases.

## Experimental procedures

### List of reagents

Chloramine-T trihydrate (Merck, Darmstadt, Germany), Copper(II)Sulphate, Penicillin, Streptomycin, RPMI-1640 (Sigma-Aldrich, Burlington, MA, USA), RPMI-1640 (Euroclone, Pero, Italy), DMEM low glucose (Sigma-Aldrich), Trypsin–EDTA (Sigma-Aldrich), Blasticidin hydrochloride (Sigma-Aldrich), Trichloroacetic acid (Merck), Fetal Bovine Serum (Merck, Darmstadt, Germany), Iodine 125 Radionuclide (Perkin Elmer, Waltham, Mass., USA), Iodogen™ iodination reagent (Pierce, Thermo-Fischer), Alexa-488 succinimidyl ester (Thermo Fischer Scientific, Waltham, Mass., USA), Anti-CD146 microbeads, anti-F4/80 microbeads, anti-CD 11b microbeads (Miltenyi Biotech, Bergisch Gladbach, Germany), HSA Alburex (CSL Behring, King of Prussia, Penn., USA), Fetal Bovine Serum (Biowest, Nuialle, France), Resazurin (biotechne, Minneapolis, Minn., USA), Liberase™TM (Roche, Basel, Switzerland), Human fibronectin was extracted from expired human plasma donated from the hospital (University Hospital of Northern Norway, Tromsø, Norway) blood-bank, by affinity chromatography locally, using the method of Vuento 1979^61^, Formaldehyde treated Serum Albumin (FSA) was prepared as described in Mego 1967^62^, Blomhoff 1984^32^, AGE-BSA was prepared as described in Hansen 2002^50^, Oxidized Low Density Lipoprotein (oxLDL) was prepared by Copper Sulphate oxidation as previously described in Li 2011^35^.

### Production and characterisation of oxHSA

Oxidation of HSA was carried out as described by Iwao 2006^24^; 300 µM HSA was incubated with 100 mM Chloramine-T in oxygen saturated PBS at 37 °C for 1 h. Afterwards the oxHSA was dialyzed against pure water and kept frozen until use. HPLC separation on Superdex-200 10/300 (Amersham Pharmacia Biotech, Amersham, UK) size exclusion column was performed. Showing that the oxHSA eluted as three peaks of 837.4, 382 and 138.4 kDa (Figure S1).

### Radiolabeling of oxHSA, FSA, AGE-BSA, oxLDL

oxHSA or FSA was radiolabeled using carrier free 125-Iodine (Perkin-Elmer) according to the Iodogen™(Pierce) method and free iodine separated from protein by PD-10 (Cytiva) desalting column, as previously described Blomhoff 1984^32^. Specific activity was calculated from amount of added protein and measured activity post-labeling.

### Animals

C57Black/6JRj mice were ordered from Janvier, and kept at the Department of Comparative Medicine, the Faculty of Health Sciences at UiT The Arctic University of Norway, under standard conditions with water and chow (SSniff, regular chow diet) ad libitum. Mice were between 8–14 weeks old for all of the procedures. All procedures were approved by the national animal research authority under the food safety administration (Mattilsynet). All animal procedures were performed in accordance with national and local guidelines, and are reported in accordance with ARRIVE guidelines.

### Method of euthanasia, anaesthesia and analgesia

Animals were euthanized by cervical dislocation, for in vivo experiments animals were anesthetized with isoflurane gas anaesthesia, and for experiments involving manipulation beyond tail vein injection, given 0.1 mg/kg buprenorphine subcutaneously at least 15 min prior to experiments, for analgesia.

### In vivo clearance, organ- and hepatocellular distribution

In vivo clearance, organ- and hepatocellular distribution was carried out as described in Santamaria-Simon 2014^63^. Briefly anesthetized mice were given intravenously 2–6 µg ^125^I labelled oxHSA for biodistribution and hepatocellular distribution. For clearance blood samples were taken from the tail, in 2–5 µL volumes over 30 min, TCA precipitation was done to quantify intact/degraded ligand. For hepatocellular distribution animals were euthanized 5 min post-injection, and cells isolated as described in the section “Isolation of primary murine liver sinusoidal endothelial cells, Kupffer cells, hepatocytes”. Organ associated activities were measured on the Perkin-Elmer Wizard^2^, blood sample and isolated cell associated activities were measured on the Packard Cobra II auto-gamma.

### Isolation of primary mouse liver sinusoidal endothelial cells, Kupffer Cells, hepatocytes

Primary mouse LSEC, KC or HC were isolated as previously described in Elvevold 2022^64^. Briefly livers were perfused and digested with 1.2 mg/50 mL Liberase TM™(Roche) centrifuged to separate hepatocytes from non-parenchymal cell fraction, and followed by immune magnetic separation (MACS, Miltenyi) of LSEC and KC from the non-parenchymal fraction by CD-146 and F-4/80, CD-11b respectively.

Primary cells were cultured in serum-free RPMI-1640 (Euro-Clone/Sigma) supplemented with 10,000 U/mL Penicillin, 10 mg/mL Streptomycin, 1:100 (Sigma).

### HEK293 cells stably expressing stabilin 1 or 2

HEK293 cells were obtained from ATCC, HEK293 expressing mouse stabilin-1 or -2, were kindly provided by Dr Sophie Johansson (University of Uppsala, Sweden)^35^, vector control cells were transfected locally by lipofectamine using the empty vector pEF6V5His-TOPO (Merck). Transfected HEK293 were grown in DMEM low glucose (Sigma) supplemented with 10,000 U/mL Penicillin, 10 mg/mL Streptomycin, 1:100, (Sigma) 7% FBS (BioWest), and 10 µg/mL Blasticidin hydrochloride for selection (Merck)^65^.

### Affinity chromatography

oxHSA, native HSA and FSA were coupled to cyanogen bromide activated Sepharose 4B (Pharmacia) as described in McCourt 1999^27^. Lysates from 19 million isolated LSEC were passed through the affinity columns, in 0.1% Triton TX-100 in PBS, columns were extensively washed with 0.1% TX-100/PBS and 0.1 M Acetic acid pH 3, 0.01 M EDTA.

Gel material was heated to 75 °C in SDS, and sent for mass spectrometry analysis^66^.

### Endocytosis experiments

Cells were seeded on human fibronectin-coated 48 well plates at 300 K cells/ well for LSEC, 300 K cells for hepatocytes, 125-320 K cells per well for KC depending on isolation yield, and allowed to adhere for 2 h before use for LSEC, KC or 4 h for hepatocytes, HEK cells were used after growing to confluence. For endocytosis experiments cells were kept in serum-free media with 1% native HSA (Alburex, CSL Behring) in RPMI-1640 (Euro-Clone) for LSEC, KC, hepatocytes and DMEM low glucose (Sigma) for HEK cells. Approximately 20,000 cpm of labelled ligand, corresponding to approximately 5–15 ng protein, was added to each well and cells were incubated for 2 h (LSEC, KC, Hepatocytes) 4 h (HEK cells) or a time course of 2, 4, 6, 18 h (LSEC). After which cell associated, non-degraded and degraded fractions were collected and measured as described in Blomhoff 1984^32^.

Briefly, culture media and one wash with PBS were collected, and acid insoluble radioactivity precipitated by addition of an equal volume of 20% trichloroacetic acid and centrifugation, half of the supernatant or acid soluble radioactivity was transferred to measure the degraded fraction. Cells were dissolved using 1% SDS, to measure cell associated radioactivity. For competitive inhibition experiments several concentrations of non-radioactive ligand containing media were added to the cells immediately prior to addition of radiolabeled ligand. Iodine-125 measurements were done using the Cobra II auto-gamma (Packard).

### Fluorescent microscopy

oxHSA and FSA were labelled with Alexa488 using the manufacturer’s instructions (Thermo Fischer). Briefly labelling reagent was dissolved in DMSO and added to a 10 mg/mL solution of protein in 0.1 M bicarbonate buffer pH 8.3 for 1 h at room temperature, then dialyzed against PBS in a 10 K MWCO Slidealyzer dialysis cassette to remove uncoupled dye.

Cells were pre-stained with Cell Mask Orange (ThermoFischer) 1:1000 for 5 min, before addition of 10 µg/mL Alexa-oxHSA for 30 min, after which cells were washed in PBS before being viewed under the EVOS (ThermoFischer) fluorescent light microscope.

### Scanning electron microscopy

Cells were seeded on human fibronectin covered glass 16 well plates at 25-40 K cells/well and allowed to attach for 2 h prior to treatment. Cells were treated with given concentrations of oxHSA in RPMI the indicated times and subsequently fixed with McDowell’s fixative. Cells were post-fixed with 1% OsO4 and dried with a graded series of ethanol (30, 60, 90, 100%) washes and finally hexamethyldisilane. Cells were sputter coated with Au/Pd immediately prior to scanning.

Scanning electron microscopy was performed the Zeiss Gemini or Sigma scanning electron microscopes at the advanced microscopy core facility at UiT.

### Viability experiments

Cell viability was assessed by LDH assay (Promega) or resazurin-resorufin (biotechne) assay were performed according to manufacturers instructions. For LDH LSEC were seeded 300 K cells/well in a 48 well plate and treated with varying concentrations of oxHSA. After set timepoints the supernatants were collected and analyzed. For resazurin-resorufin cells were seeded the same way, with 1:10 resazurin reagent (biotechne) added to the culture media and measurements, using the ClarioStar plate reader wavelengths excitation 530–570 nm emission 580–590 nm, done at 3 and 6 h.

### Supplementary Information

Supplementary Information 1.

Supplementary Information 2.

## Data Availability

The datasets used and/or analyzed during the current study available from the corresponding author on reasonable request.
