# Supplementary material for: Highly oxidized albumin is cleared by liver sinusoidal endothelial cells via the receptors stabilin-1 and -2
Source: Sci Rep. 2023 Nov 5;13:19121. doi: 10.1038/s41598-023-46462-9 (PMC10625979; doi:10.1038/s41598-023-46462-9)

**Supplemental Figures**

**Title:**Highly oxidized albumin is cleared by liver sinusoidal endothelial cells via the receptors stabilin -1 and -2.

**Author Names:** Christopher Holte*^1^, Karolina Szafranska^1^, Larissa Kruse^1^, Jaione Simon-Santamaria^1^, Ruomei Li^1^, Dmitri Svistounov^2^, & Peter McCourt^1^.


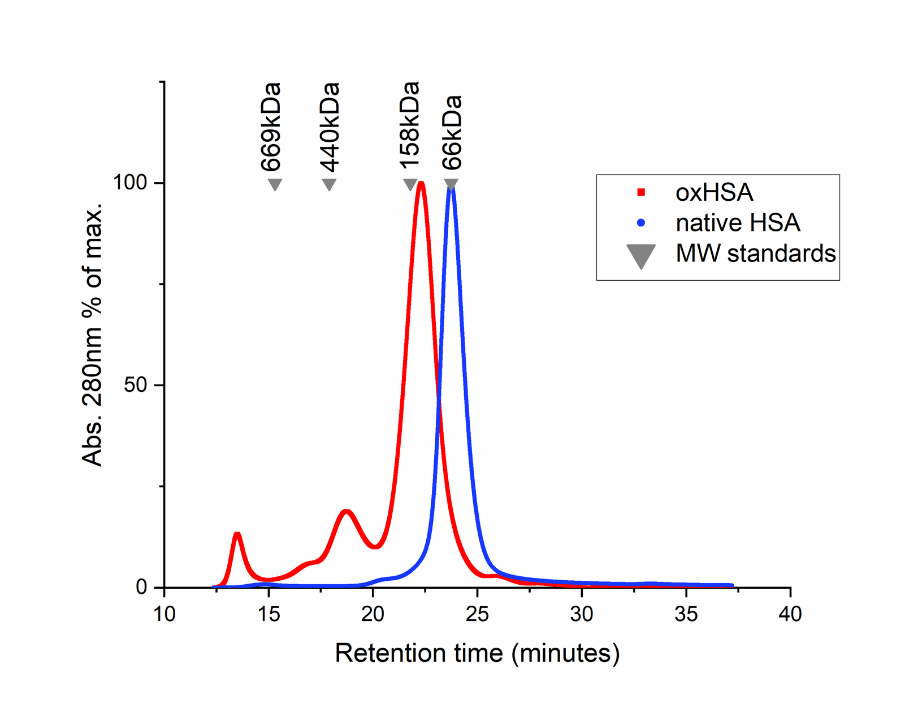


**Figure S1:** **Supedex-200 10/300 HPLC chromatogram** of oxHSA (red) superimposed on chromatogram of native HSA (blue), MW standards are indicated with gray triangles. X-axis: retention in minutes. Y-axis: absorbance at 280nm as % of maximum.


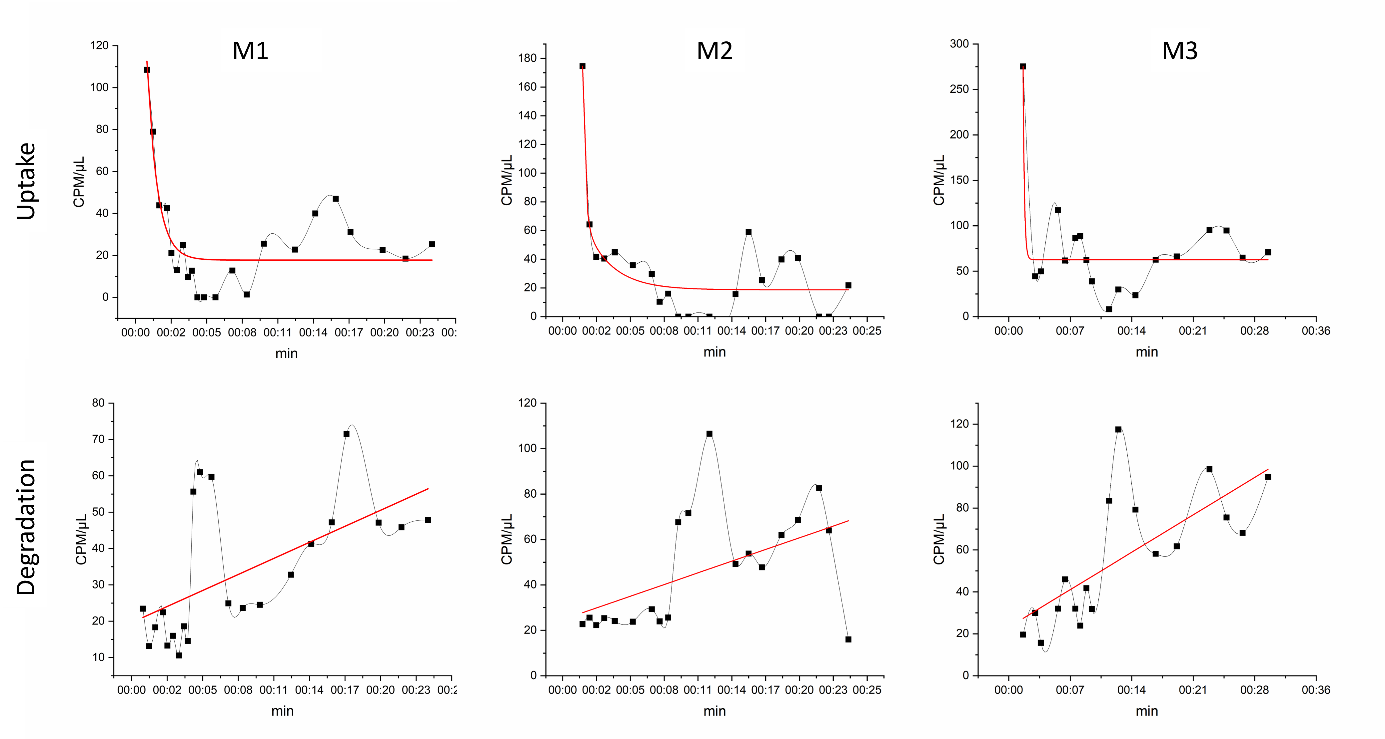


**Figure S2: Blood clearance curves** from biodistribution study. X-axis: time in minutes, y-axis: CPM/µl blood. 3 mice were injected with 1-5µg ^125^I labelled oxHSA, and blood samples collected 0-30minutes post-injection, each animal is plotted separately. Top panels: Removal of acid insoluble (intact protein) radiation from blood. Bottom panels: appearance of acid soluble radiation (degraded protein).

**
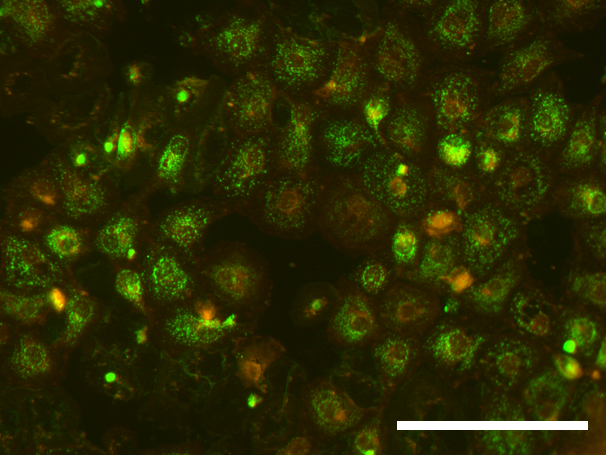
**

**Figure S3:** **Fluorescence micrograph** of LSEC given Alexa488 labelled oxHSA (10µg/mL x 30min). Cell membranes were stained with Cell Mask Orange 1:1000 x 5 min before addition of oxHSA. oxHSA staining shows expected vesicular pattern. Scale bar = 100µm.


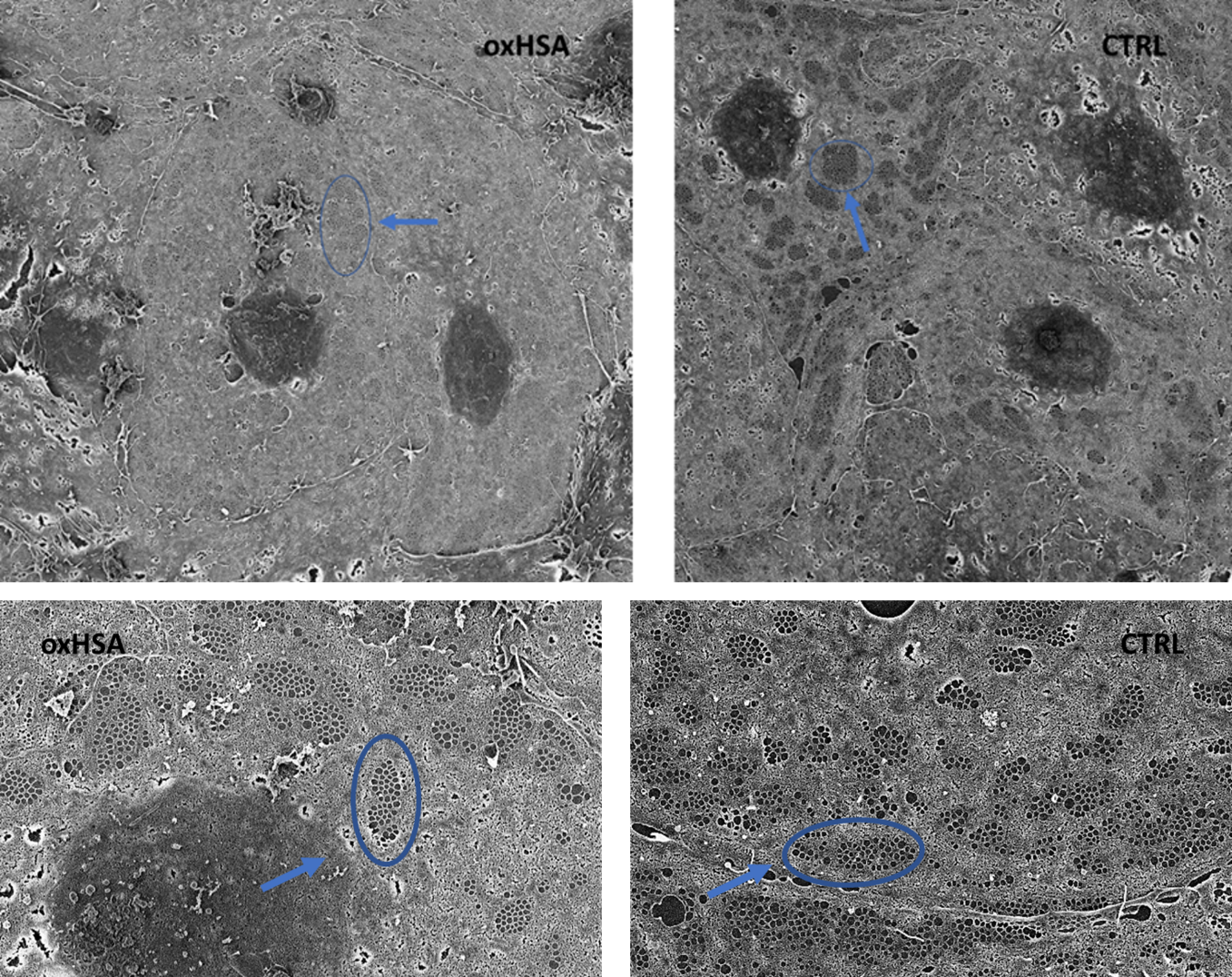


**Figure S4:** **Scanning Electron Micrographs** of LSEC treated with 160µg/mL oxHSA (**oxHSA**) and untreated controls (**CTRL**). Representative images, overview/large FOV (top) or close-up (bottom), of LSEC treated with 0 (CTRL) or 160µg/mL oxHSA (oxHSA) for 1 hour. Arrows and ellipses indicate fenestrations organized into sieve-plates.


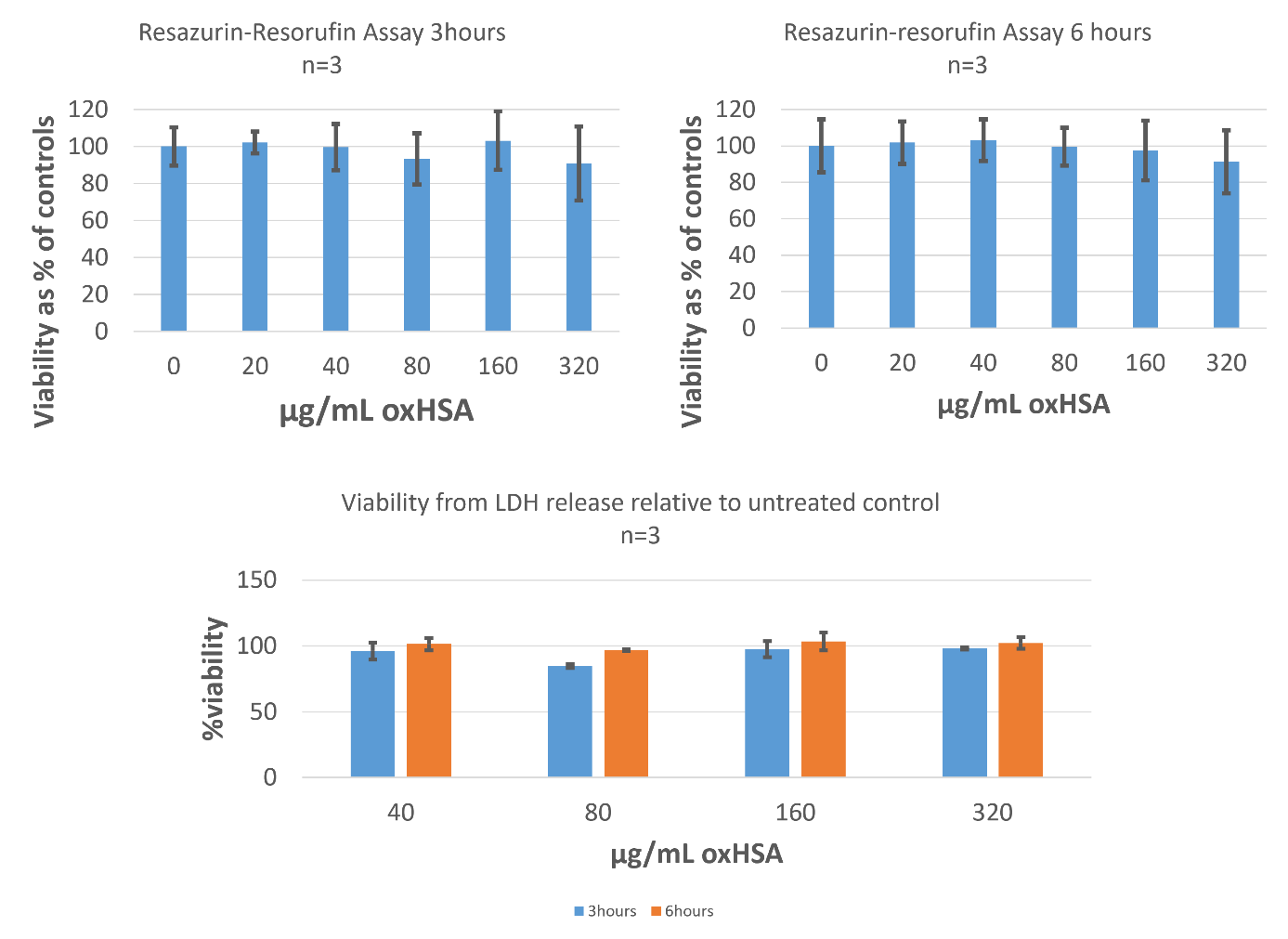


**Figure S5:** **Viability assays** performed on LSEC. TOP PANNELS Resazurin-resorufin assay: 300K cells were treated with indicated concentrations of oxHSA with resazurin added to the culture media, measurements were done at 3 and 6 hours after addition of oxHSA. BOTTOM PANELS LDH-Glo assay: 300K cells were treated with indicated concentrations of oxHSA, and supernatant samples collected at 3 and 6 hours. Viability calculated from LDH release relative to positive control (triton x-100) and negative control (untreated cells).

**Table S1: Mass spectrometry** data of MW range of stabilins 1 and 2 for affinity column extracted lysates, in descending order: oxHSA column, Dummy (without protein) column, and native albumin column. Complete MS data is included in separate supplementary files.


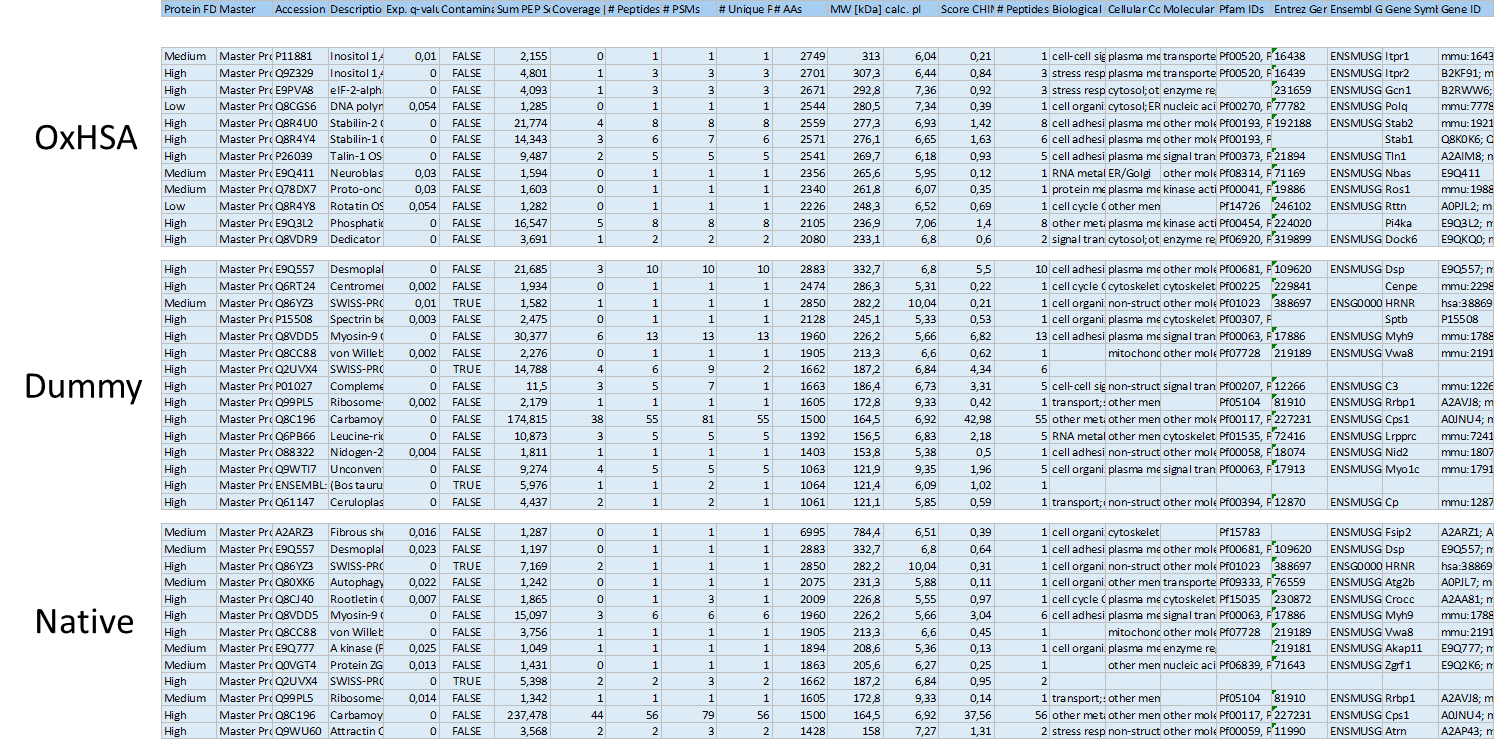

Supplement: Supplementary file 2 — Supplementary Information 2. [file 41598_2023_46462_MOESM2_ESM.docx]
